# Dominant malaria vector species in Nigeria: Modelling potential distribution of *Anopheles gambiae* sensu lato and its siblings with MaxEnt

**DOI:** 10.1371/journal.pone.0204233

**Published:** 2018-10-03

**Authors:** Godwin E. Akpan, Kayode A. Adepoju, Olakunle R. Oladosu, Samuel A. Adelabu

**Affiliations:** 1 African Regional Centre for Space Science and Technology Education in English (ARCSSTEE), Obafemi Awolowo University (OAU), Ile-Ife, Osun State, Nigeria; 2 Department of Geography, University of The Free State, Qwaqwa Campus, Qwaqwa, Phuthaditjhaba, South Africa; Hitit University, Faculty of Medicine, TURKEY

## Abstract

Malaria is a major infectious disease that still affects nearly half of the world’s population. Information on spatial distribution of malaria vector species is needed to improve malaria control efforts. In this study we used Maximum Entropy Model (MaxEnt) to estimate the potential distribution of *Anopheles gambiae* sensu lato and its siblings: *Anopheles gambiae* sensu stricto, and *Anopheles arabiensis* in Nigeria. Species occurrence data collected during the period 1900–2010 was used together with 19 bioclimatic, landuse and terrain variables. Results show that these species are currently widespread across all ecological zones. Temperature fluctuation from mean diurnal temperature range, extreme temperature and precipitation conditions, high humidity in dry season from precipitation during warm months, and land use and land cover dynamics have the greatest influence on the current seasonal distribution of the *Anopheles* species. MaxEnt performed statistically significantly better than random with AUC approximately 0.7 for estimation of the *Anopheles* species environmental suitability, distribution and variable importance. This model result can contribute to surveillance efforts and control strategies for malaria eradication.

## Introduction

*Anopheles* species have plagued the world with malaria for decades and centuries now. An estimated 3.2 billion people worldwide were at risk of malaria in 2014 [1]. In 2016, ninety-one (91) countries and territories in the world had ongoing malaria transmission with estimated 216 million cases of malaria and 445,000 malaria deaths [2]. Fifteen countries accounted for 80% of all malaria cases and deaths globally. Sub-Saharan Africa region was home to 90% of malaria cases and 91% of malaria deaths, globally. Nigeria accounted for the highest proportion of cases globally (27%), followed by the Democratic Republic of the Congo (10%), India (6%) and Mozambique (4%) [2].

Besides *Anopheles funestus*, *Anopheles gambiae* is the dominant and most efficient vector of human malaria in the Afrotropical Region [3–5], based on its high abundance, longevity, high propensity for humans feeding, and high vectorial capacity [6,7]. *Anopheles gambiae* complex herein referred to as *Anopheles gambiae* sensu lato (s.l.) is made up of eight reproductively isolated species that are almost indistinguishable morphologically: *An*. *arabiensis*, *An*. *gambiae* sensu stricto (s.s.), *An*. *bwambae*, *An*. *melas*, *An*. *merus*, *An*. *quadriannulatus*, *An*. *coluzzii*, and *An*. *amharicus* [8,9]. Environmental, social and demographic factors such as climate change/variability drive the distribution of the dominant malaria vector species and their parasite transmission [10,11]. They shift in response to changes in temperature and precipitation [12–15]. Onyabe and Conn [4] explained that climatological factors, especially total annual precipitation strongly influence the range and relative abundance of *An*. *arabiensis* and *An*. *gambiae* within forest zones and savannas [16–18], with *An*. *arabiensis* predominating during the dry season and *An*. *gambiae* becoming more abundant during the rainy season [19]. In line with recent studies [20,21], Onyabe and Conn [4] observed shifts in species composition of *An*. *arabiensis* and *An*. *gambiae* s.s. after two years in four of 10 localities in their study, attributing it to random temporal (seasonal) fluctuations. Also, Umar *et al*. [22] observed low population of female *Anopheles* mosquitoes during the dry seasons (January to June and October to December), while carrying out assessment of indoor resting density of female anopheline mosquitoes in human dwelling at malaria vector sentinel sites in Bauchi State, Nigeria. According to Umar *et al*. [22], higher densities of anopheline mosquitoes during the rainy season to a large extent explains the seasonal pattern of clinical cases of malaria, with peak transmission shortly after maximum annual rainfall [19,23].

An understanding of the temporal and spatial determinants of parasite transmission, its seasonal patterns and the dominant vectors implicated in transmission is crucial [24] for the control of vector species [25]. A reliable risk modelling is one of the precautionary means in the framework of public health and management of malaria, especially in view of climate change [26]. Numerous mathematical models have been applied for disease risk modelling [27–30]. Maximum entropy algorithm (MaxEnt)—a type of machine learning technique of ecological niche modelling has been proved to perform well in modelling the distribution of disease vectors and their possible disease transmissions including that of malaria [28–30]. MaxEnt aims to predict potential distribution of biological species from the observation of species occurrences [31,32]. Moffett *et al*. [28] used MaxEnt to construct niche models for 10 malaria vector species in Africa, and predicted that *An*. *gambiae* abundance was highest in West Africa followed by *An*. *arabiensis*, *An*. *funestus* and *An*. *melas*; with human population density as the critical factor determining malaria risk. Similarly, Kulkarni *et al*. [30] used MaxEnt in Northern Tanzania when they found seasonality of precipitation and maximum annual temperature to have contributed the most to niche models for *Anopheles arabiensis* and *An*. *funestus* s.l. with AUC of 0.989 and 0.991 respectively, cold season precipitation and elevation were also found important for *An*. *gambiae* s.s. with AUC of 0.997.

While studies on MaxEnt modelling of the *Anopheles* species [28–30] predicted potential distribution on all spatial locations within geographic area of interest, without considering ecological zones. The identification of the species distribution based on entomological surveys [18–22] considered the species presence in different ecological zones with respect to absolute locations rather than all spatial locations of interest. In this study, we used MaxEnt for modelling environmental suitability and potential distribution of these dominant malaria mosquitoes in all spatial locations across topographic relief, ecological and regional zones in Nigeria. We also assessed the contributions of bioclimatic and other environmental variables to the occurrence of these *Anopheles* species.

## Materials and methods

### Study area

**Fig 1:**
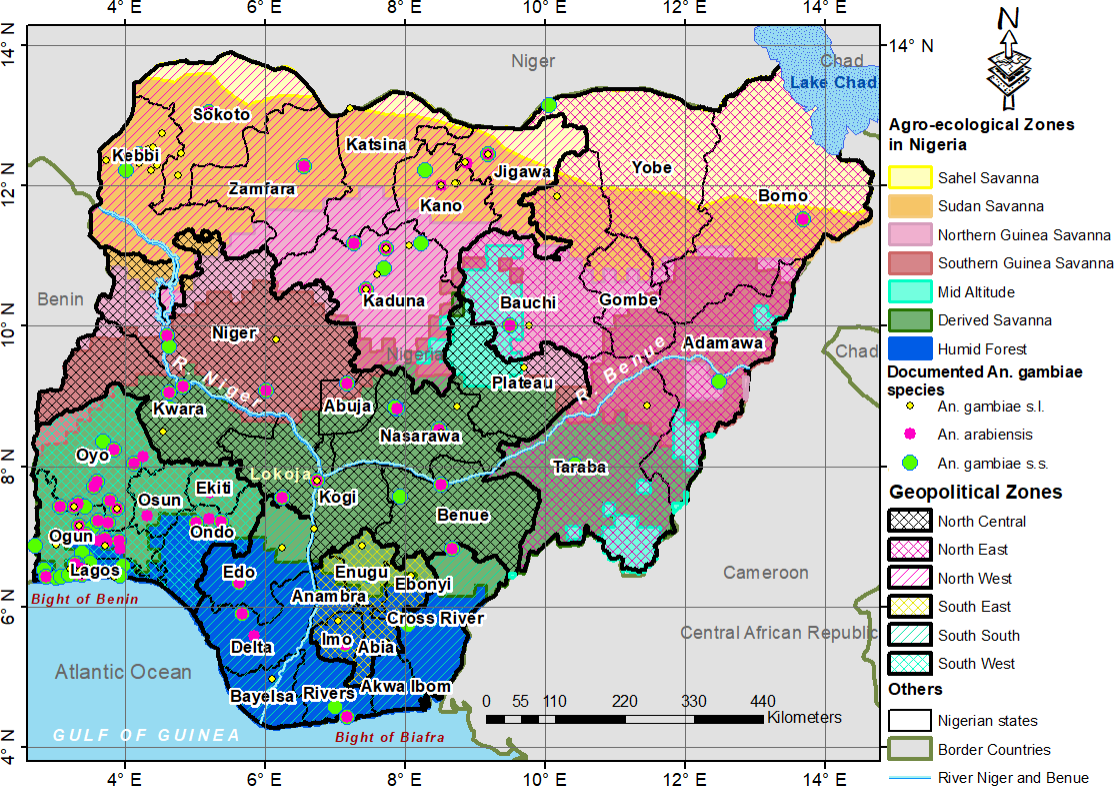
Study area with documented points of *Anopheles gambiae* species. Georeferenced *Anopheles* species locations reprinted for illustrative purposes only from Okorie *et al*. [39] under a CC BY 4.0 license, with permission from PLOS ONE.

Nigeria is a country in West Africa, located approximately between Latitudes 4^o^ and 14^o^ north of the Equator and between Longitudes 2^o^ 2' and 14^o^ 30' east of the Greenwich Meridian (Fig 1) [33]. It has a total area of 923.77km^2^ characterised by undulating topographic relief, patterned by valleys created by its river systems (Fig 1) [33,34]. Coastal plains in the south have mean elevation of about 150m above sea level. Northern plains rise to about 600-700m, with Jos Plateau (over 1,500m) within Nigeria’s geographic centre, and Mambilla plateau (over 2,100m) amongst mountains at the border with Cameroon [35,36]. Temperature varies across ecological zones (Fig 1). Tropical at the coast (within Humid forest and Derived savanna) with 10°C and 37°C extreme low and high temperatures respectively, sub-tropical further inland (within Derived and Guinea savannas), and semi-arid in the far north (within Sudan and Sahel savannas) with 6°C and 44°C extreme low and high temperatures respectively [17,34]. Mid Altitude zone of Jos and Mambilla plateaus has average monthly temperatures range of 21–25°C [34]. Annual rainfall ranges from 500mm to 750mm in the north, and 1,200mm to above 4000mm in the south [34]. This diversity in climate conditions across the country affects the spatial epidemiology of malaria mosquitoes, malaria transmission and human vulnerability [24]. About 90% of over 190 million Nigerians are at the risk of malaria [37,38].

### Modelling procedures and data analysis

#### Data resources

Malaria vectors data (Occurrence data: 1900–2010) was obtained from Nigeria *Anopheles* vector database (S1 Table); a comprehensive review of Okorie *et al*. [39]. Also, 19 bioclimatic variables (1960–1990) with about 1km^2^ spatial resolution were obtained from WorldClim—Global Climate Data (http://www.worldclim.org), global climate models (GCM)—community climate system model version 4 (CCSM4) [40] to model the impact of current climates on malaria vector species distribution in Nigeria. Additional variables used include land use land cover data with 24 classes obtained from U.S. Geological Survey data release [41], and Digital Elevation Model (DEM) derived from Shuttle Radar Topography Mission (SRTM) 90m obtained from the Consultative Group on International Agricultural Research—Consortium for Spatial Information (CGIAR-CSI) [42].

#### Model operation

Model operation was carried out as implemented in Maximum entropy algorithm model (MaxEnt) version 3.3.3k, described in detail previously by Phillips *et al*. [43]. All environmental layers were modified in ArcMap to be at the same extent, and a bias layer was created to provide MaxEnt with background samples to guard against bias in datasets [44]. Locations where *Anopheles* species were sampled to occur was defined as 1; the remaining pixels that were not sampled had “no data” in the grid [45]. Taking advantage of all available data without having an independent dataset, occurrence data for each species was split twenty-one times into training (75%) and testing (25%) subsets under sub-sample replicated run type. This was done to test the model performance (robustness and predictiveness) given by Area Under the Receiver Operating Characteristic (ROC) Curve or AUC; a plot of sensitivity against specificity which measures the ability of the model to discriminate between sites where a species is present (***y = 1***) against where it is absent (***y = 0***) [46–48]. Maximum iterations was increased from 500 (default) to 5000, allowing the model to have adequate time for convergence guarding against over-prediction or under-prediction of the relationships by the model [47]. Other setting options were left at default including regularization of 1 that helped reduced model over-fitting.

Probability of species occurrence predicted by all the predictor environmental variables produced a point-wise mean (model images) [47]. This was classified in ArcMap for current distributions of *Anopheles gambiae* s.l. and its siblings. This was also classified into suitable and unsuitable habitats with 10 percentile training presence logistic threshold provided by MaxEnt. Suitable areas within ecological zones for the studied mosquito species were generated by MaxEnt based on the entropy of optimal climatic and other environmental conditions that match the empirical average (threshold value) within documented species records. The 10% minimum threshold meant that suitable habitat was defined to include 90% of the data used to develop the model, considering some errors the data used may likely had [47]. Zonal statistics in ArcGIS was used to determine the average distribution density of predicted *Anopheles* species in all ecological and geopolitical zones, and in each state. Distribution density less than or equal to one (≤1) defines the probability of the species not occurring, greater than one (>1) defines species presence, while 2 represents maximum prevalence of species within a state and each zone. Analysis of percent contribution of each variable and jackknife test of variable importance were used to examine the contributions of environmental variables (Table 1) in defining the *Anopheles* species suitable habitats [30]. Jackknife test shows the training gain of each variable if the model was run in isolation, and compare it to the training gain with all the variables [47]. Comparing three jackknife plots produced for training gain, test gain and AUC gave very informed evaluations of variables contributions [46].

**Table 1:** Environmental variables used.

Code	Bioclimatic/Ecological variables
BIO1	Annual Mean Temperature
BIO2	Mean Diurnal Range (Mean of monthly (max temp—min temp))
BIO3	Isothermality (BIO2/BIO7)*100
BIO4	Temperature Seasonality (standard deviation *100)
BIO5	Maximum Temperature of Warmest Month
BIO6	Minimum Temperature of Coldest Month
BIO7	Temperature Annual Range (BIO5-BIO6)
BIO8	Mean Temperature of Wettest Quarter
BIO9	Mean Temperature of Driest Quarter
BIO10	Mean Temperature of Warmest Quarter
BIO11	Mean Temperature of Coldest Quarter
BIO12	Annual Precipitation
BIO13	Precipitation of Wettest Month
BIO14	Precipitation of Driest Month
BIO15	Precipitation Seasonality (Coefficient of Variation)
BIO16	Precipitation of Wettest Quarter
BIO17	Precipitation of Driest Quarter
BIO18	Precipitation of Warmest Quarter
BIO19	Precipitation of Coldest Quarter
LULC_NIG	Land use Land cover of Nigeria
DEM_NIG	Digital Elevation Model of Nigeria (Land Surface Terrain)

Note

* denotes multiplication.

## Results

### Potential suitable areas for the occurrence and distribution of *An*. *gambiae* s.l. and its siblings

**Fig 2:**
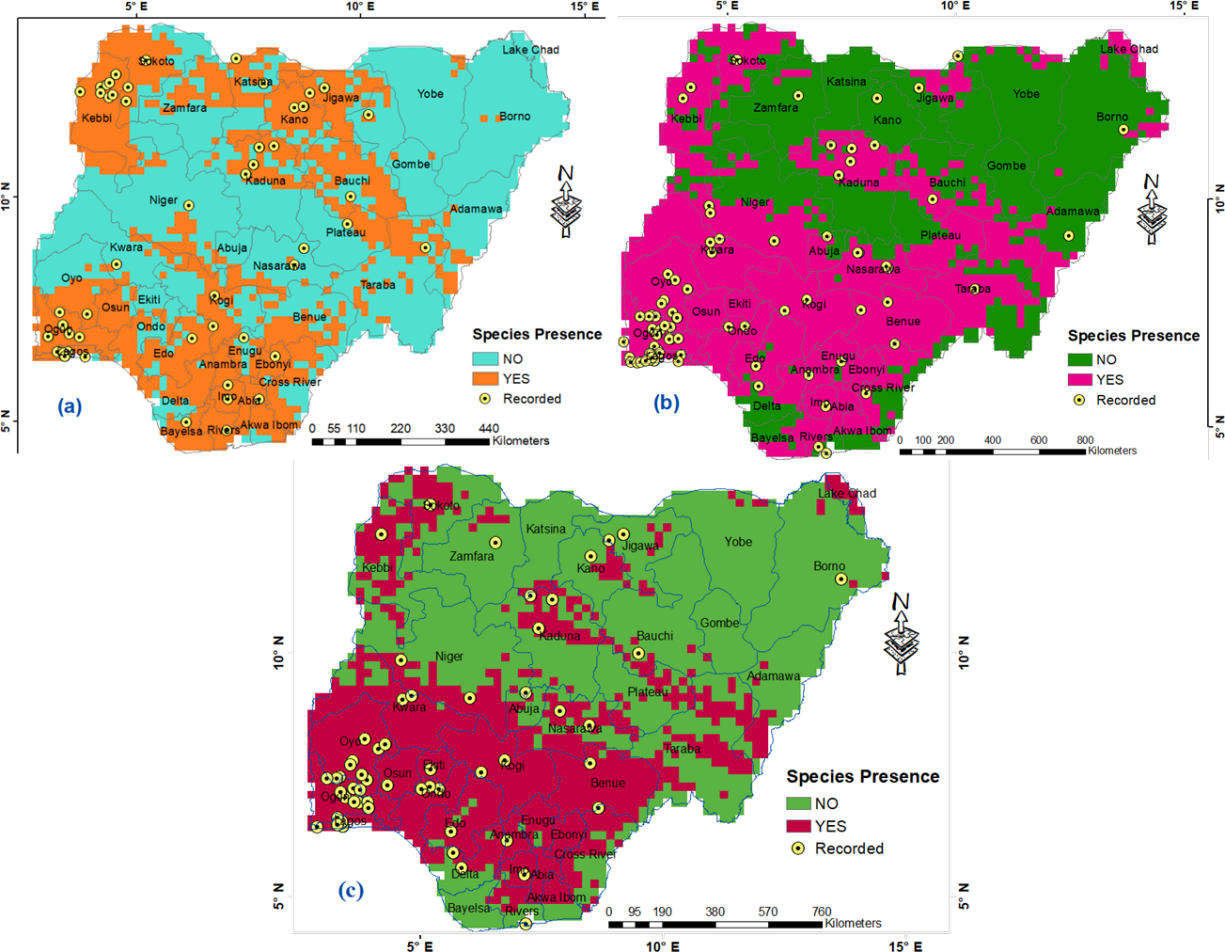
**Suitable habitats for *Anopheles* species in Nigeria: (a) *An*. *gambiae* s.l., (b) *An*. *gambiae* s.s. and (c) *An*. *arabiensis*.** Georeferenced *Anopheles* species locations reprinted for illustrative purposes only from Okorie *et al*. [39] under a CC BY 4.0 license, with permission from PLOS ONE.

The result of MaxEnt modelling predicted that the approximately 85,000 square kilometre (km^2^) Humid forest and 204,000km^2^ Derived savanna are highly suitable for the occurrence and distribution of *An*. *gambiae* s.l., *An*. *gambiae* s.s. and *An*. *Arabiensis*; with prevalence between 65% and 71% (Fig 2; Table 2). This makes all the states in South West, South East, South South and parts of North Central regions within the two ecological zones highly suitable for the *Anopheles gambiae* species (Fig 2; Table 2). The estimated 25,000 km^2^ Mid Altitude zone of Jos and Mambilla plateaus, and highlands along boundary with Cameron (Fig 2) seem less suitable with prevalence between 57% and 59% (Table 2), just as parts of Mangrove and Fresh water swamp forests within the Humid forest, especially along the deltas within Delta, Bayelsa, Rivers and Akwa Ibom states (Fig 2). Also, highlands within Niger, Kwara, Oyo, Ondo, Ekiti, Edo, Kogi, Enugu and Anambra states appear less suitable for these species especially *An*. *gambiae* s.l. Moreover, Sudan savanna and parts of Northern Guinea savanna in the North Western region are highly suitable for the occurrence of *An*. *gambiae* s.l. (Table 2), while North Eastern region landmass seems less suitable (Fig 2A). Sokoto and Kebbi states in the North West within Sudan savanna are highly suitable for the occurrence of *An*. *gambiae* s.s. and *An*. *arabiensis*, just as Kaduna South / Kaduna Central and parts of Jigawa, Kano and Zamfara states (with large less suitable landmass). Similar to *An*. *gambiae* s.l., landmass in Sahel, Sudan, Northern and Southern Guinea savannas within North Eastern region appear less suitable for *An*. *gambiae* s.s. and *An*. *Arabiensis* (Fig 2; Table 2). However, areas within Lake Chad, Bama and Kala/Balge districts of Borno state appear suitable for *An*. *gambiae* s.s. and *An*. *arabiensis*, just as Yobe state boundary with Niger Republic, and considerable landmass from Taraba, Adamawa, Gombe, through Plateau to Bauchi state (Fig 2).

**Table 2:** Ecological zones suitability and distribution of *Anopheles* species.

Ecological zone	Estimated Area (km^2^)	Mean Distribution Density per sq. km			Prevalence (%)		
		*An*. *gambiae* s.l.	*An*. *gambiae* s.s.	*An*. *arabiensis*	*An*. *gambiae* s.l.	*An*. *gambiae* s.s.	*An*. *arabiensis*
Sahel savanna	80,277.79	1.19	1.15	1.12	59.38	57.71	56.12
Sudan savanna	152,500.02	1.34	1.18	1.16	67.21	58.96	58.08
Northern Guinea savanna	91,944.45	1.24	1.20	1.17	61.96	60.22	58.57
Southern Guinea savanna	109,444.46	1.20	1.24	1.20	60.06	61.88	59.82
Mid Altitude	25,000.00	1.17	1.18	1.16	58.45	59.02	57.90
Derived savanna	204,166.69	1.30	1.39	1.36	65.18	69.26	68.24
Humid forest	85,277.79	1.43	1.35	1.36	71.47	67.59	67.79

**Note:** distribution density = 1km^-2^ is equivalent to prevalence = 50% (unsuitable zone and species absence, designated with a green square); >1km^-2^ ≡ >50% (suitable zone and species presence); and 2km^-2^ ≡ 100% (highly suitable zone with maximum prevalence of species, designated with a red square).

**Fig 3:**
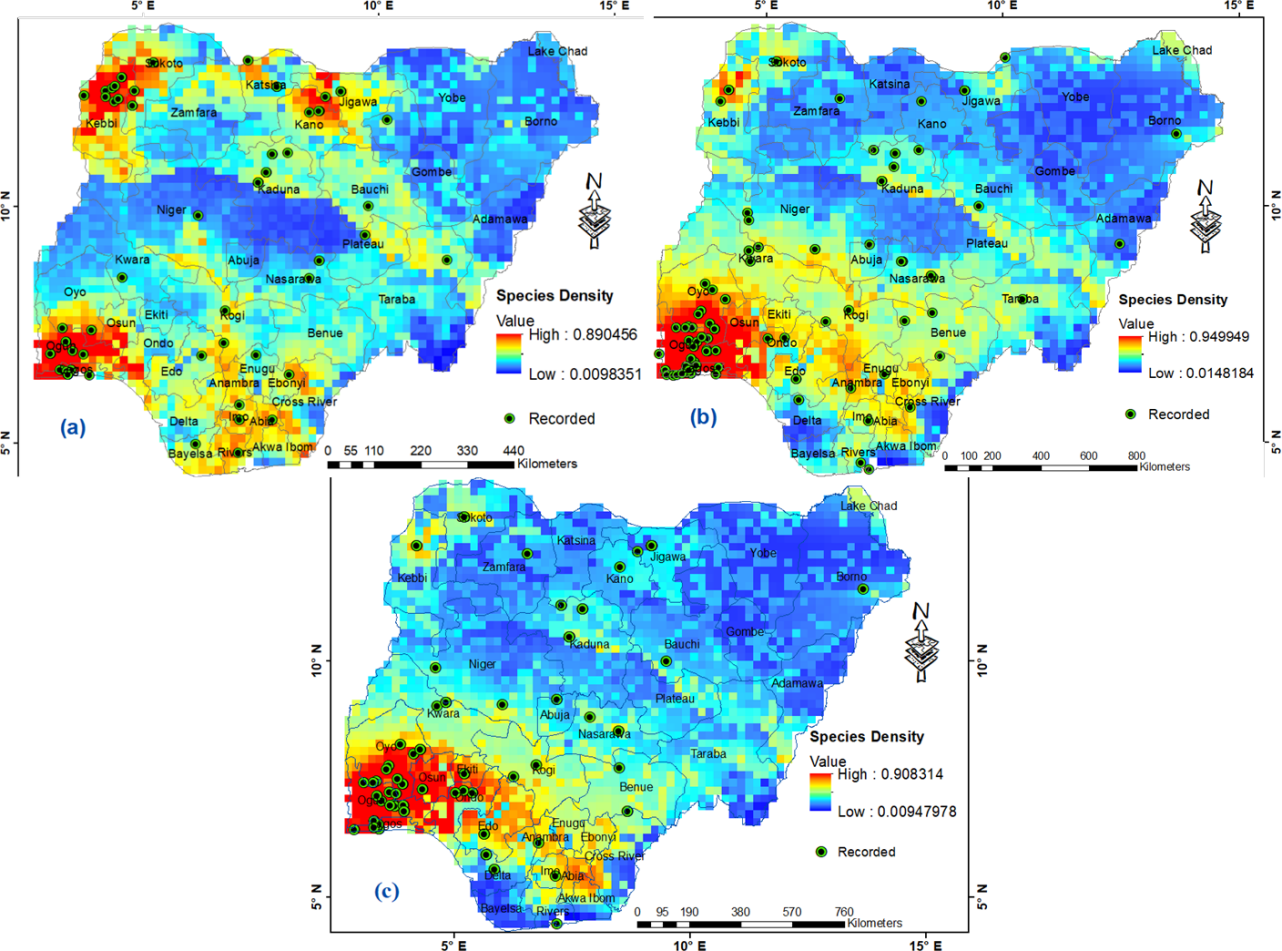
**Potential distribution of *Anopheles* species in Nigeria: (a) *An*. *gambiae* s.l., (b) *An*. *gambiae* s.s. and (c) *An*. *arabiensis*.** Georeferenced *Anopheles* species locations reprinted for illustrative purposes only from Okorie *et al*. [39] under a CC BY 4.0 license, with permission from PLOS ONE.

**Fig 4:**
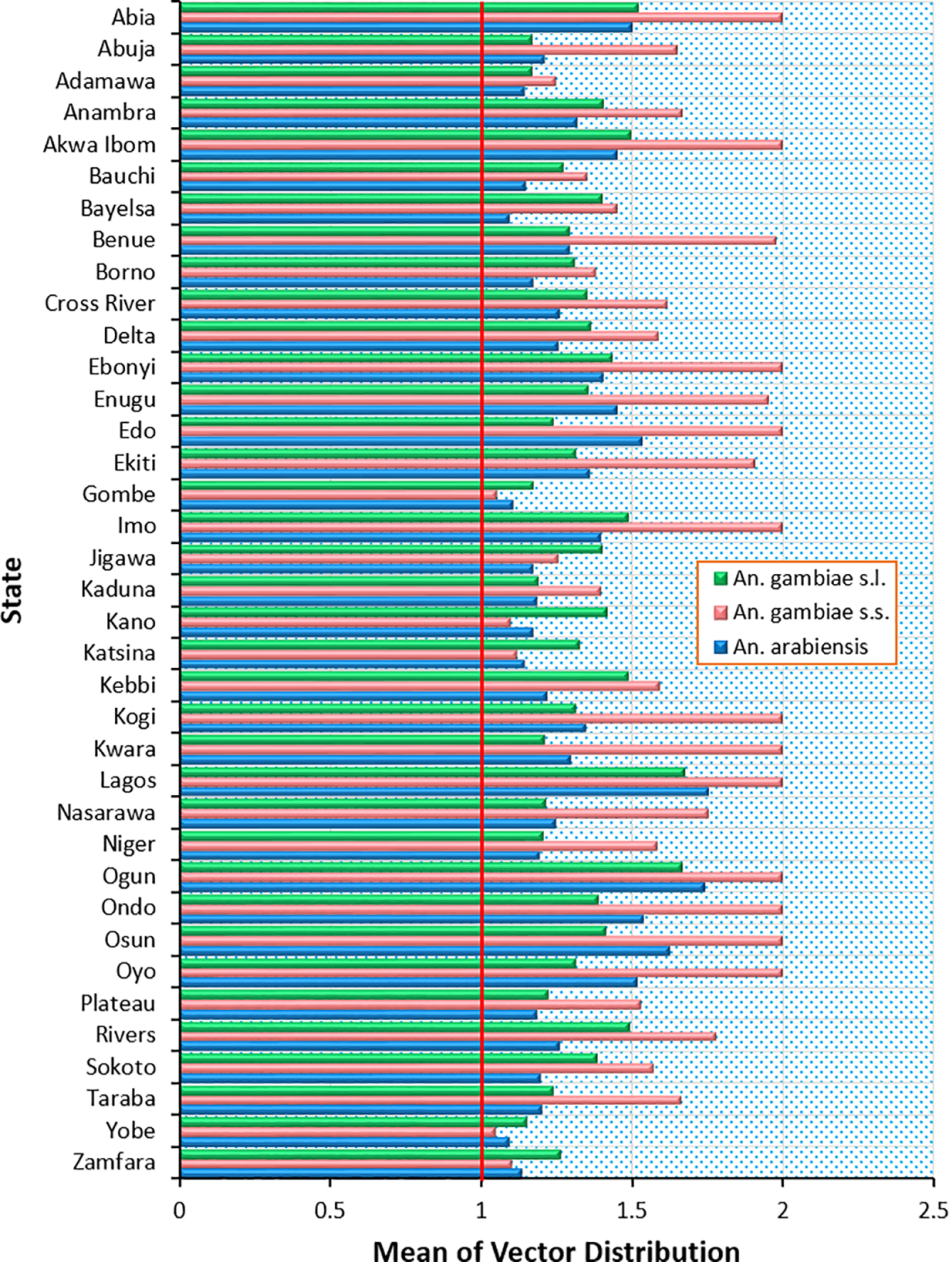
Mean distribution density of *An*. *gambiae* s.l., *An*. *gambiae* s.s., and *An*. *arabiensis* in Each Nigerian State. Note: if 1 ≡ 0 (species do not occur); then >1 = species occur; and 2 = maximum species prevalence.

With respect to their environmental suitability, *An*. *gambiae* s.l., *An*. *gambiae* s.s. and *An*. *arabiensis* are widespread in Humid forest, Derived savanna; less distributed in Northern and Southern Guinea savannas, and least distributed in Sahel savanna and Mid Altitude zones (Table 2; Fig 3). While *An*. *gambiae* s.l. is widespread in Sudan savanna, *An*. *gambiae* s.s. and *An*. *arabiensis* record high presence only within west of Sudan savanna (Table 2; Fig 3). Unlike *An*. *gambiae* s.l., *An*. *gambiae* s.s. and *An*. *arabiensis* with similar distribution pattern are widespread within Lake Chad region of Sahel savanna, but record limited presence in Fresh water and Mangrove swamp forests of the Niger Delta region (Fig 3). In terms of regional zones, *An*. *gambiae* s.l. is more widespread in the South East, followed by South West and South South (Table 3); with highest mean distribution density in Lagos followed by Ogun and Abia States, while Yobe state records the lowest among all states (Figs 3A and 4). North East has the lowest mean distribution density of *An*. *gambiae* s.l. (Table 3). The boundary line on the mean distribution density graph defines presence and absence condition for each *Anopheles* species from zonal statistics (Fig 4). *An*. *gambiae* s.s. is highly prevalent in South Western and South Eastern regions of Nigeria (Table 3; Fig 4). It is more widespread in North Central region than South South and North West, and lowest in North East (Table 3). As a dominant *Anopheles* species [6,7], *An*. *arabiensis* exists in all states in Nigeria, highest in Lagos state and lowest in Bayelsa state (Fig 4). South West records highest prevalence of *An*. *arabiensis*, followed by South East, South South, North Central, North West, and lowest in North East (Table 3).

**Table 3:** *Anopheles* species prevalence by regions under current climate.

Geopolitical zone	Estimated Area (km^2^)	Mean Distribution Density per sq. km.			Prevalence (%)		
		An. gambiae s.l.	An. gambiae s.s.	An. arabiensis	An. gambiae s.l.	An. gambiae s.s.	An. arabiensis
South South	68,888.90	1.38	1.29	1.29	69.10	64.46	64.65
South East	23,611.11	1.44	1.42	1.41	71.94	71.12	70.75
South West	61,666.67	1.43	1.57	1.59	71.29	78.28	79.70
North Central	186,388.91	1.24	1.30	1.25	61.75	64.85	62.51
North East	227,500.02	1.20	1.17	1.14	59.79	58.48	57.05
North West	177,222.24	1.34	1.20	1.18	67.16	60.07	58.95

**Note:** distribution density = 1km^-2^ is equivalent to prevalence = 50% (species absence, designated with a green square); >1km^-2^ ≡ >50% (species presence); and 2km^-2^ ≡ 100% (maximum prevalence of species, designated with a red square).

Variability exists in the spatial distributions of the *Anopheles* species (Fig 3) among states (Fig 4) across topographic relief, ecological and geopolitical regions based on the diversity in climate conditions across the country, which affects the spatial epidemiology of these *Anopheles* species and malaria transmission [24]. Matching the pattern of malaria parasite prevalence [25], distribution density of the *Anopheles* species increases from the sub-tropical Middle Belt region to the tropical southern regions with high rainfall and coastal plains (Fig 3). Especially South West and South East where Lagos state, smallest by landmass but the most populated, and most urbanised [39,49] records highest prevalence of *An*. *gambiae* s.l., *An*. *gambiae* s.s. and *An*. *arabiensis*, matching about 1.2 million confirmed cases of malaria in 2016 [50].

### Environmental variables contributions in defining *Anopheles gambiae* species distributions

**Fig 5:**
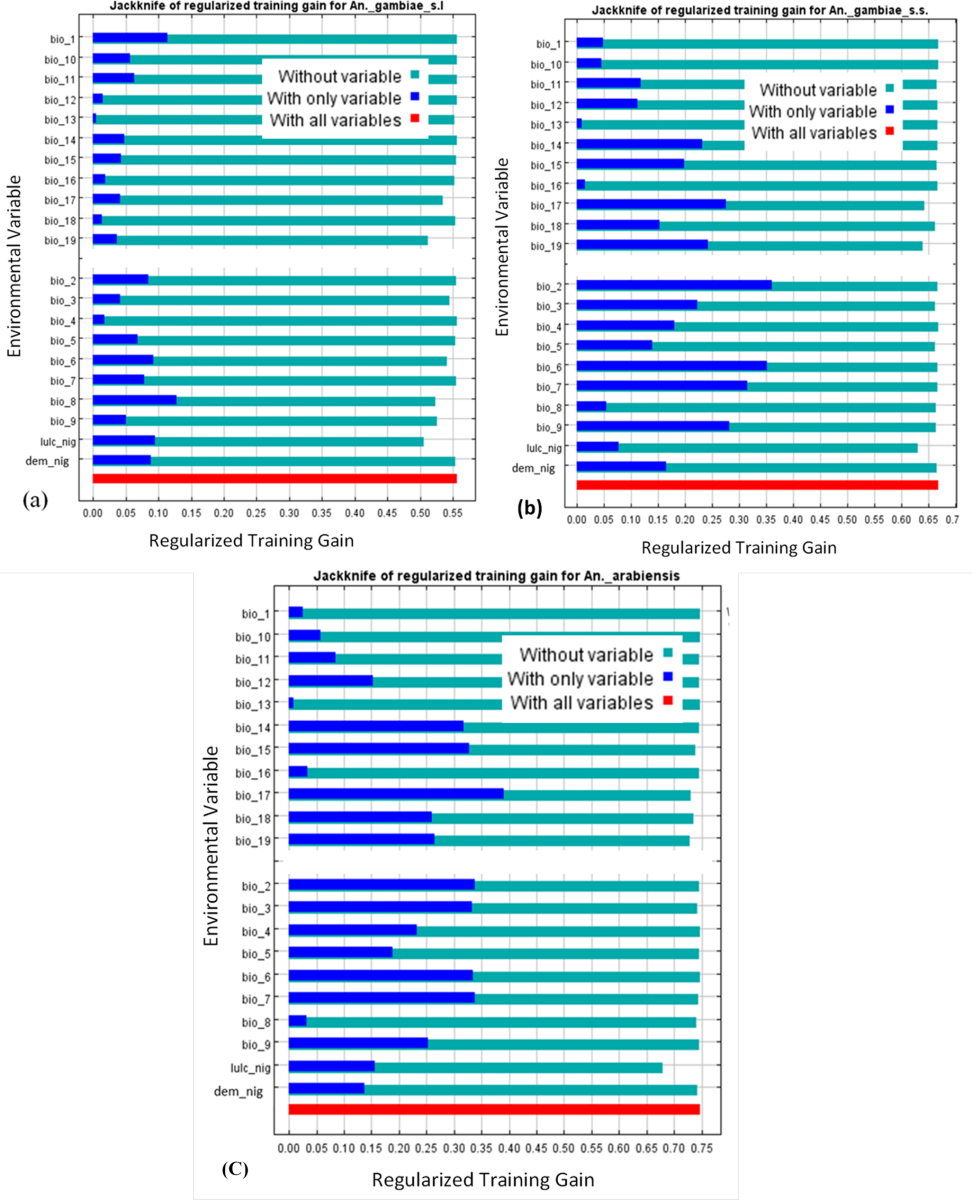
Jackknife test for (a) *An*. *gambiae* s.l. (b) *An*. *gambiae* s.s. (c) *An*. *Arabiensis*.

Mean temperature of wettest quarter (bio_8) is the environmental variable with highest gain to the MaxEnt model of *An*. *gambiae* s.l. when used in isolation, which therefore appears to have the most useful information by itself (Fig 5A). Bio_8 demonstrates how mean temperatures (28°C minimum and 30°C maximum) [51] during the wettest three months (June—August) of the year may affect seasonal distributions of *An*. *gambiae* s.l. Other environmental variables that influence the occurrence and distribution of *An*. *gambiae* s.l. when used together with all other environmental variables are minimum temperature of coldest month (bio_6) (23°C, August), precipitation of coldest quarter (bio_19) (June—August, about 211mm in the arid north to above 2000mm in the coastal south), annual mean temperature (bio_1) 33°C, and precipitation of driest quarter (bio_17) (December—February, 0mm in arid north to 240mm in coastal south) (Fig 5A) [52]. Also, mean diurnal range (bio_2) (7–16°C) [52] is the major environmental variable which defines suitable habitats for *An*. *gambiae* s.s. in isolation, and constitutes the relevance of temperature fluctuation on spatial distribution of *An*. *gambiae* s.s. Other variables which appear very pivotal in the occurrence and distribution of *An*. *gambiae* s.s. when used alongside all other environmental variables are minimum temperature of coldest month (bio_6), temperature annual range (bio_7) (28–36°C), mean temperature of driest quarter (bio_9) (20°C minimum and 35°C maximum), precipitation of driest quarter (bio_17), precipitation of coldest quarter (bio_19) and precipitation of driest month (bio_14) (January, not exceeding 27mm in the wet southern coast) (Fig 5B) [51,52]. Precipitation of driest quarter (bio_17) is the major environmental variable that influences the occurrence and seasonal distributions of *An*. *arabiensis* in isolation (Fig 5C). Precipitation of coldest quarter (bio_19), precipitation seasonality (bio_15), mean diurnal range (bio_2), mean temperature of driest quarter (bio_9) and precipitation of warmest quarter (bio_18) (March to May, about 10mm in the arid north to 700mm in the coastal south) combine with all other environmental variables to delineate suitable habitats for the occurrence and distribution of *An*. *arabiensis* (Fig 5C). However, land use land cover map reflecting high urbanisation, increased population density and anthropogenic activities is the environmental variable that would decrease the gain the most for all three *Anopheles* species if omitted. It has the most information that is not present in the other variables. The critical influence of land use land cover dynamics in the occurrence and distribution dynamics of *An*. *gambiae* s.l., *An*. *gambiae* s.s. and *An*. *arabiensis* is expressed in their high distribution density in highly populated/urbanised states with increased anthropogenic activities including Abia, Akwa Ibom, Anambra, Enugu, Imo, Kano, Lagos, Ogun, Ondo, Osun, Oyo, Rivers and Sokoto states (Fig 4) [13].

### MaxEnt model performance

MaxEnt recorded a fair performance for 21 replicate runs of *An*. *gambiae* s.l., *An*. *gambiae* s.s., and *An*. *arabiensis* with average test AUC of 0.713, 0.699, and 0.713 respectively. The value of AUC determines the performance of the model; AUC of 0.5 implies that the model was no better than random, while an AUC of 1 indicates a perfect prediction. In essence, AUC values tend to be higher for species with narrow ranges relative to the study area described by the environmental data. A behaviour that is an artifact of the AUC statistic, but does not necessarily mean that the models are better [46].

## Discussion

In agreement with previous studies [4,17,23,39], the model results suggested that *An*. *gambiae* s.l., *An*. *gambiae* s.s., and *An*. *arabiensis* are widespread across all ecological zones in Nigeria, where they co-exist in sympatric relationship [24]. The combinations of soil, landform and climatic characteristics within ecological zones define distinct distribution of the modelled *Anopheles* species [36], and they predominantly occur in Humid forest, Guinea savannas and Sudan savanna regions [4,39,53–56]. The high environmental suitability of the Derived savanna and Humid forest within southern and parts of North Central regions [28,39] is influenced by human settlement patterns, topographical and climatic conditions of the regions [11,28,57]. Total annual precipitation, random temporal fluctuations, climate seasonality and land use land cover dynamics strongly influenced the range, relative abundance and ecological adaptability of the dominant members of the *An*. *gambiae* complex, in line with previous findings [4,16,28,30]. The highest mean distribution density of *An*. *gambiae* s.s. amongst other species corroborates with the results of Bruce-Chwatt [17] and Okwa *et al*. [18] who reported *An*. *gambiae* s.s. as the most efficient and most widespread within the *gambiae* complex [58]. Its high abundance is highly associated with the mean diurnal temperature range that increases the species sensitivity to changes in climates, leading to widespread presence of the species [52,59]. In accordance with Oyewole *et al*. [19], the combined contributions of environmental variables favour higher distribution of *An*. *gambiae* s.s. in wet season than *An*. *arabiensis*, while precipitation during dry and warm months (high humidity in dry season) favour higher distribution of *An*. *arabiensis* than *An*. *gambiae* s.s. in dry season [20,21,30]. *An*. *arabiensis* preference of warmer climates [19,30] possibly impacts its limited presence in the cold swamps within the Humid forest, and the high suitability of Sahel savanna localities, especially Lake Chad basin area of Borno state enhanced by high relative humidity from the lake [53].

The low suitability of areas within Mid Altitude zone may be attributed to the comparatively cold weather of the highlands associated with average monthly temperatures 21–25°C [14,34], relatively below the model optimum temperatures of 23–35°C for rapid population expansion of the *An*. *gambiae* species [12]. As derived from the model, the high suitability of Lagos state for the malaria vector species can also be attributed to high temperature and precipitation in that area of the Humid forest [12]. The complex nature of the society, poor planning and lack of infrastructure in expanding slum areas, rapid population expansion and industrial activities make the climate warmer and create conditions highly suitable for malaria vector reproduction, survival and increased biting rates, exacerbating malaria transmission in Lagos state [11,14,28]. The influence of seasonal rainfall variability and high tropical temperatures on the extent and unbalanced distribution of the modelled mosquito species observed in most part of the country also agrees with the findings of Oyewole *et al*. [19] and Umar *et al*. [22], in relation to unbalanced and seasonal malaria transmission. The observed gradient in the distribution density from coastal south to arid north, shows that vector abundance is greatest in areas with consistently high temperatures and in any case, small mean diurnal temperature range and consistent precipitation. This is in line with the observation of Dimitrov and Morton [52] who reported that entomological inoculation rate was highest in the coastal areas and lowest in the northeast.

MaxEnt performance was better than random [45] with AUC values less than those obtained in similar studies [28,30]. This may be attributable to large ranges of the documented species [46] relative to the study area (especially in the North Eastern part of the country), resulting in increased sampling bias [44,60,61], which may influence the model performance [46,47]. However, according to Lobo *et al*. [62], an accurate model for widespread species (just as the ones modelled in this study) where the probability of presence increases steadily with predictor values have low AUC values, denoting the true generalist nature of the species distribution. It is important to note that suitable areas with low distribution density of the modelled *Anopheles gambiae* species may likely experience widespread prevalence with high distribution density, species migration and invasion [63], if there is a change in any of the environmental variables identified in this study as crucial to their distribution pattern. This will lend credence to the prediction of ecological models, that the distribution of world biomes is likely to shift as a result of changes in climate system associated with increased warming [64], since *An*. *gambiae* s.l., *An*. *gambiae* s.s., and *An*. *arabiensis* highly flourish with warm climate [12,65]. Thus, the propensity of future malaria transmission in Nigeria is expected to be higher with seasonal spatial shifts due to climate change and altered weather patterns; influencing the range (both latitude and altitude), intensity, and seasonality of vectors [11–15,65,66].

## Conclusions

In this paper we used Maxent in modelling environmental suitability and distribution of dominant *Anopheles gambiae* species in Nigeria. We also assessed the contributions and importance of bioclimatic and other environmental variables to the model. Results showed that the species are more prevalent within the Humid forest and the Derived savanna, but most prevalent in South Western and South Eastern geopolitical zones within the two ecological zones. This is particularly worrisome in highly populated and urbanised Lagos state which recorded highest distribution density of all three species. Our results also showed that land use dynamics become very critical for the occurrence and distribution of the three dominant species of *Anopheles*, while seasonal rainfall, temperature fluctuations and high humidity during warm weather (dry season) drive the occurrence and seasonal distribution of the *Anopheles* species and potential malaria transmission. The derived MaxEnt model was successful in defining potential suitable habitats for the occurrence and distribution of the *Anopheles* species, and estimated variable importance. This result might be useful in predicting the variability of malaria vector distribution across ecological gradients and in understanding the potential causes of its severity from an environmental point of view in tropical regions.

## Supporting information

S1 Table Nigeria *Anopheles* vector database. Reused from Okorie *et al*. [39] under a CC BY 4.0 license, with permission from PLOS ONE.
(XLSX)
